# Social signals and aversive learning in honey bee drones and workers

**DOI:** 10.1242/bio.021543

**Published:** 2016-11-28

**Authors:** Arian Avalos, Eddie Pérez, Lianna Vallejo, María E. Pérez, Charles I. Abramson, Tugrul Giray

**Affiliations:** 1 Carl R. Woese Institute for Genomic Biology, University of Illinois at Urbana-Champaign, Urbana, IL 61801, USA; 2 Department of Biology, University of Puerto Rico, San Juan, PR 00931, USA; 3 Department of Mathematics, University of Puerto Rico, San Juan, PR 00931, USA; 4 Department of Psychology, Oklahoma State University, Stillwater, OK 74074, USA

**Keywords:** Honey bee, Alarm pheromone, Drone, Semiochemicals, Associative learning, Social communication

## Abstract

The dissemination of information is a basic element of group cohesion. In honey bees (*Apis mellifera* Linnaeus 1758), like in other social insects, the principal method for colony-wide information exchange is communication via pheromones. This medium of communication allows multiple individuals to conduct tasks critical to colony survival. Social signaling also establishes conflict at the level of the individual who must trade-off between attending to the immediate environment or the social demand. In this study we examined this conflict by challenging highly social worker honey bees, and less social male drone honey bees undergoing aversive training by presenting them with a social stress signal (isopentyl acetate, IPA). We utilized IPA exposure methods that caused lower learning performance in appetitive learning in workers. Exposure to isopentyl acetate (IPA) did not affect performance of drones and had a dose-specific effect on worker response, with positive effects diminishing at higher IPA doses. The IPA effects are specific because non-social cues, such as the odor cineole, improve learning performance in drones, and social homing signals (geraniol) did not have a discernible effect on drone or worker performance. We conclude that social signals do generate conflict and that response to them is dependent on signal relevance to the individual as well as the context. We discuss the effect of social signal on learning both related to its social role and potential evolutionary history.

## Abstract (precis)

**Summary:** Alarms serve to focus attention and prioritize individual concerns. In honey bees, social alarm impairs appetitive learning. Here, we show that the effect on aversive learning depends on social role and signal intensity.

## INTRODUCTION

Social structures rely on communication between individual members of a group (Alaux et al., 2010; Johnson and Linksvayer, 2010). Group level responses such as defense, and resource gathering and allocation, are critically dependent on the ability of individual members to convey their perceived experiences to others (Seeley, 1995; Johnson and Linksvayer, 2010). Much like cells in an organism, individual worker honey bees (*Apis mellifera*) respond to nest mate signals to better coordinate responses to environmental stimuli. This system inherently induces conflict within perceiving honey bees, as a response must be evaluated for both its individual and social consequence. In addition, the signal may not carry the same meaning to all members of the colony, i.e. worker castes versus reproductive castes (e.g. queens, drones). In this study we begin to explore this conflict, focusing on how social alarm (e.g. alarm pheromone component, IPA) modulates a specific individual response within sterile social workers and male reproductive (drone) honey bees.

The perception of signals inherently establishes conflict for an individual. Receivers can benefit from the information, but only in the correct context as the attention and possible action which the signals demand may be in opposition. One example can be found in the mountain spiny lizard, *Sceloporus virgatus*; here, the perceived availability of receptive females modulates male escape behavior to the point where his likelihood of escaping possible predation is greatly reduced (Cooper and Wilson, 2007; Cooper, 2009). Given the complexity of communication in the honey bee colony, bees are an excellent model through which conflicts inherent in the perception of signals can be explored at the level of the social structure.

For social communication, insect colonies rely on a suite of signaling mechanisms that make complex interactions possible (Johnson and Linksvayer, 2010; Wilson, 1965). Like other social insects, honey bees (*Apis mellifera* sp.) use a variety of methods including food exchange, vibration, and olfaction to communicate colony needs (Michelsen et al., 1986; Nieh, 2010; Seeley, 1989, 1995, 1997; Seeley et al., 2012). The more prevalent method of social communication is the use of volatile chemical compounds (Ali and Morgan, 1990; Alaux et al., 2010; Ayasse et al., 2001; Slessor et al., 2005; Wilson, 1965). These compounds stimulate the coordination of individual behaviors into a consensus capable of achieving tasks critical to the colony. So integral and complex is pheromone communication, that it is often considered analogous to the endocrine signaling of metazoans (Alaux et al., 2010; Billen and Morgan, 1998; Slessor et al., 2005; Wilson and Sober, 1989; Wilson, 1965). Ultimately, pheromones functionally regulate socially critical aspects of honey bee behavior such as caste differentiation, colony defense, and resource localization (Ali and Morgan, 1990; Le Conte and Hefetz, 2008; Slessor et al., 2005).

Within honey bee pheromones, the functional effects of a broad variety of the component chemicals have been characterized (Allan et al., 1987; Collins and Blum, 1982; Collins and Rothenbuhler, 1978; Le Conte and Hefetz, 2008; Koeniger et al., 1979; Pickett et al., 1981; Slessor et al., 2005; Williams et al., 1981). Indeed, the analysis of complete pheromone cocktails, or their component chemicals on bee behavior, has been an extensive field of research for the past fifty years. This research described quantifiable behavioral responses whose genetic mechanisms are beginning to be explored (Alaux, et al., 2009; Alaux and Robinson, 2007; Ali and Morgan, 1990; Le Conte and Hefetz, 2008; Grozinger et al., 2007; Urlacher et al., 2010). Yet our understanding of how these colony-level forms of communication impact and compete at the level of individual behaviors is still nascent.

Learning assays have been used to study the social effects of pheromone presentation (Alaux and Robinson, 2007; Grozinger et al., 2003; Kocher et al., 2009). This research has shown that queen-specific pheromones generally inhibit aversive associations, while alarm pheromones directly disrupt appetitive learning (Becker et al., 2000; Urlacher et al., 2010; Urlacher et al., 2013; Vergoz et al., 2007). Indeed, the effect of alarm pheromone even seems to be evolutionarily conserved in *Apis cerana*, a close relative of *A. mellifera* (Wang et al., 2016).

Defense is a critically important component of honey bee colony survival, and alarm signals elicit fast and robust behavioral responses (Allan et al., 1987; Collins and Rothenbuhler, 1978; Koeniger et al., 1979). Work by Urlacher et al. (2010) demonstrated that exposure to either alarm pheromone or its primary component, isopentyl acetate (IPA), negatively affected the individual worker bees' ability to establish simple appetitive associations. The effect was dose-dependent, with bee performance steadily decaying as the presented dose increased, and later stabilizing at the higher dosage levels (Urlacher et al., 2010). Other work indicated that the effect is not caste-specific. Becker et al. (2000) showed a negative effect on the ability of drones to form appetitive associations when exposed to IPA.

In this study we examined if the detrimental effect of exposure to IPA on honey bee learning influences aversive as well as appetitive associations, and if these effects are similar across worker and reproductive drones. In addition, we tested how responses to IPA compare to geraniol (the primary component of the Nasonov's gland) and one non-social odor, cineole (an extract of *Eucalyptus* tree leaves).

## RESULTS

### IPA presentation influences aversive learning response in workers but not in drones

#### Dose-dependent effects of IPA on worker aversive learning

**Fig. 1.**
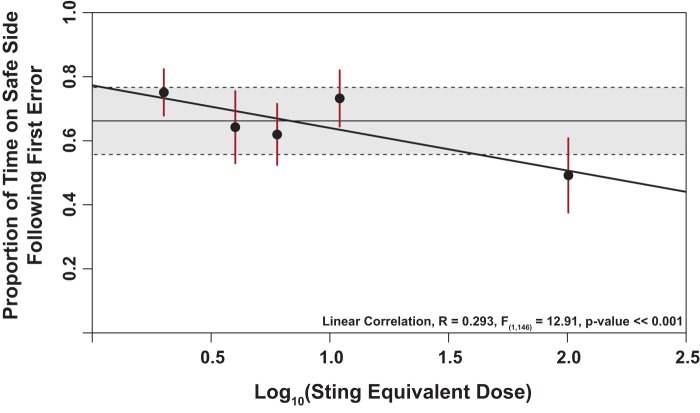
**Correlation analysis of learning response in honey bee workers under continuous exposure to increasing doses of IPA.** Here we show the correlative relationship between IPA dose exposure and response. Dots represent the group means, lines indicate each group's 95% confidence interval. The shaded region represent the mean of the control group (solid horizontal line) bounded by its 95% confidence interval (dashed lines). The solid diagonal line illustrates the linear relationship of the correlation between the log base 10 of the IPA sting equivalent dose and our response metric. Results show a highly significant negative association between the two variables. Statistical test was a linear correlation of the proportion of time on safe side following the first error and the log-transformed sting equivalent dose.

The presentation of IPA at low levels resulted in the highest aversive learning performance in workers, yet at the highest dose [100 sting-equivalent dose (SED)] learning was poor. Control bees were intermediate between the low and high dose groups. Correlational analysis of worker response showed that indeed there was a significant negative correlation of IPA on aversive learning [r=0.247, *P*=0.0014, df=1]. This correlation was robust even when we accounted for possible bias in the control group due to past experience [r=0.293, *P*=0.00047, df=1; see Fig. 1].

**Fig. 2.**
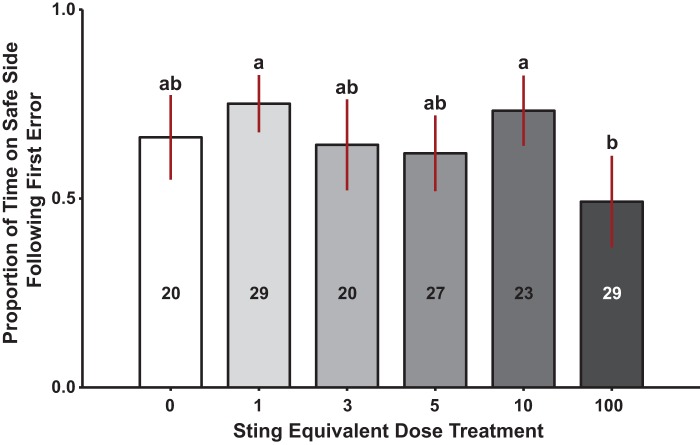
**Effect of incremental doses of continuously presented IPA on worker learning.** Performance is measured for each individual as proportion of time spent on the safe side of the apparatus during the first (acquisition) trial of aversive training following the first experience of punishment (mild shock). Bars indicate means and lines illustrate the 95% confidence interval for each group tested. Letters above the bars display statistical relationships between dosage groups. Numbers inside the bars are group sample sizes. Results show that the greatest statistical significance detected was between the 1 and 100, and the 10 and 100 Sting Equivalent Dose (SED) groups. Statistical test was a two-way analysis of variance on the logit transformed proportion of time on safe side following the first error. This was followed by a post-hoc Tukey's test of pairwise comparisons. Assessment is detailed in the ‘Data analysis’ section of the Materials and Methods.

These effects do not depend on color preferences or proximity to alarm pheromone source. Across workers, there was no statistically significant interaction between IPA dose level and shock area color [ANOVA, *F*_5,146_=0.918, *P*=0.47]. An independent simple main effect was observed for dose [ANOVA, *F*_5,146_=3.73, *P*=0.003] and a trend towards significance was observed in the shock area color [ANOVA, *F*_1,146_=3.26, *P*=0.07]; no effect was observed for distance from the lane housing the scented filter paper [ANOVA, *F*_4,146_=0.958, *P*=0.43]. Post hoc analysis via a Tukey's range test showed that no significant differences were present between the control (0 SED) and any of the treatment groups, but rather that significance was primarily being driven by differences across the dosage groups, specifically 1 SED vs 100 SED and 10 SED vs 100 SED (Fig. 2).

#### Drone aversive learning is not influenced by IPA presentation

**Fig. 3.**
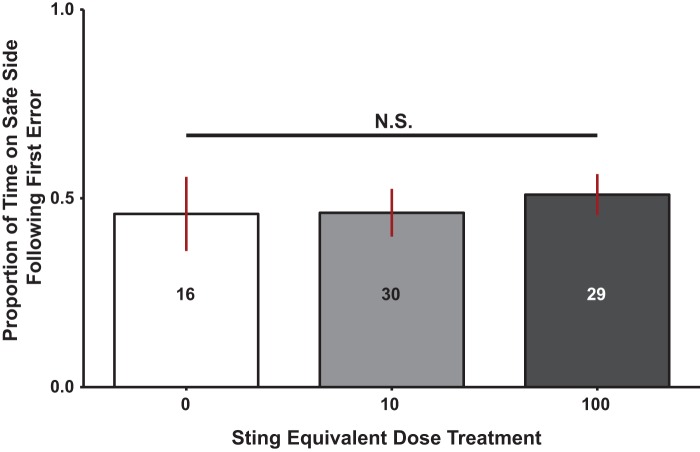
**Effect of variable dose levels of continuously presented IPA on drone response.** Provided is a summary of the statistical relationship between IPA dose groups and drone response in our learning paradigm. The Y axis represents proportion of time spent on the safe side following the first error, the X axis outlines our treatment levels of IPA sting equivalent doses. Bars represent group means, lines the 95% confidence interval. Numbers inside the bars represent group sample sizes. Results show that there were no statistically significant differences in drone performance between any of the groups.

In contrast to the workers, the two IPA dose levels did not affect performance of drones [ANOVA, *F*_2,73_=0.873, *P*=0.44] (Fig. 3). The performance of drones did not change across IPA doses, and the treatment groups were not different from control group drones.

As in workers, color preferences or proximity to the source of the alarm pheromone did not influence the performance of drones. For drones, no main interaction effect was detected between IPA dose presentation and shock area color [ANOVA, *F*_2,73_=2.288, *P*=0.11]. No statistically significant main effects were observed in performance across shock area color [ANOVA, *F*_1,73_=0.210, *P*=0.65], or distance from odor cue [ANOVA, *F*_4,73_=0.664, *P*=0.62].

### Alternative odor presentation to workers and drones demonstrate specificity of IPA effects

In contrast to the high dose of IPA that did reduce learning, the positive social pheromone, geraniol, did not influence learning performance of either workers or drones. The non-social odor cineole did not influence the learning of an aversive task in workers, but did improve the aversive learning performance of drones.

#### Aversive learning of workers exposed to 100-bee equivalent of geraniol or to similar dose of cineole

**Fig. 4.**
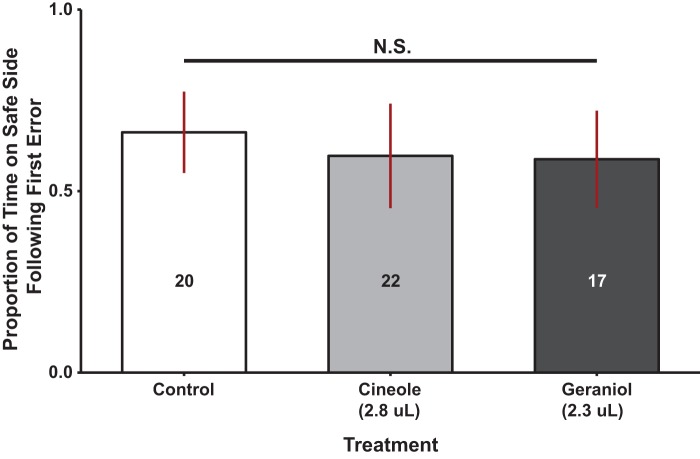
**Learning performance of workers under continuous exposure to various odor cues and signals.** The graph shows the statistical relationship of various continuously presented odors on the learning performance of honey bee workers. The Y axis shows the total time spent on the safe side of the electric shock avoidance assay following the first error. The X axis outlines the type of odor provided as well as the corresponding volume presented in brackets. Numbers inside the bars correspond to sample sizes. Results show that no significant differences in worker learning response were observed between control and treatment groups.

In workers, there was no significant main effects for either odor [ANOVA, *F*_2,57_=0.986, *P*=0.38] or shock area color [ANOVA, *F*_1,57_=0.059, *P*=0.81], and no significant interaction between the two factors [ANOVA, *F*_2,57_=0.067, *P*=0.94]. In addition, no simple main effect was detected for the distance from the lane housing scented filter paper [ANOVA, *F*_4,57_=0.729, *P*=0.58] (Fig. 4).

#### Aversive learning of drones exposed to 100-bee equivalent of geraniol or to similar dose of cineole

**Fig. 5.**
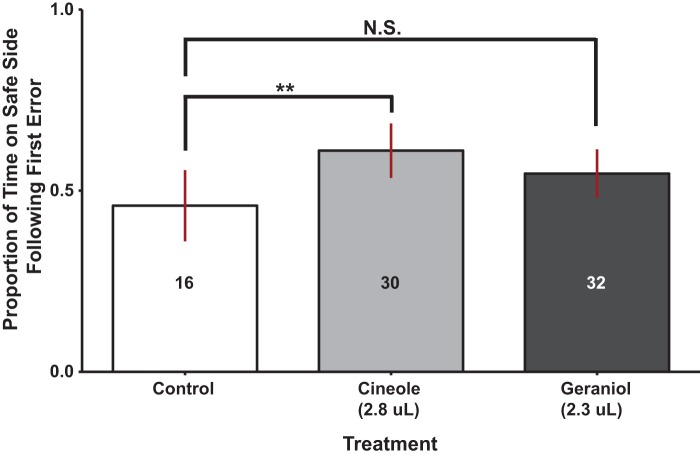
**Effects of alternate odors on drone response during a punishment with discrimination learning assay.** Here we summarize the statistical relationship in learning performance between groups under continuous exposure to various alternate odors. The Y axis represents our response metric, while the X axis provides odor presented and volume of presentation in brackets. Bars indicate sample means, lines represent the 95% confidence interval of each group. Numbers inside the bars correspond to sample sizes. Results show that drone performance was improved by the continuous presentation of cineole, a floral cue, and remained unaffected by the presentation of geraniol, a social homing signal. **P=0.023 by two-way analysis of variance on the logit-transformed proportion of time on safe side following the first error, followed by a post hoc Tukey's test of pairwise comparisons.

In contrast to the response of workers, a significant main effect was detected across odor presentations in drones [ANOVA, *F*_2,76_=3.626, *P*=0.03]. Post hoc analysis revealed that the observed differences were primarily due to a significant effect between the control and cineole treatment groups (Fig. 5). In drone's response, no bias towards shock area color [ANOVA, *F*_2,76_=0.734, *P*=0.48] or distance from the odor cue [ANOVA, *F*_4,76_=0.995, *P*=0.42] was detected, and no significant interaction was observed between odor and shock area color [ANOVA, *F*_2,76_=0.734, *P*=0.48].

## DISCUSSION

The most important conclusion of this study is that influence of a social signal is dependent on the information it conveys (alarm versus homing), the type of learning, and the social role of the recipient. IPA did not impair aversive learning. The effect was dose-dependent with high performance at low doses and low performance at the highest dose in workers, and no discernible effect evident in drones (Figs 1, 3 and 5). In addition, a social homing signal (geraniol) did not have a noticeable effect in drone or worker performance (Figs 4 and 5) in aversive learning as opposed to reports in the literature suggesting improved performance in appetitive learning (Urlacher et al., 2010). Interestingly, cineole, a non-social odor, improved the learning performance in drones in an aversive learning assay.

**Fig. 6.**
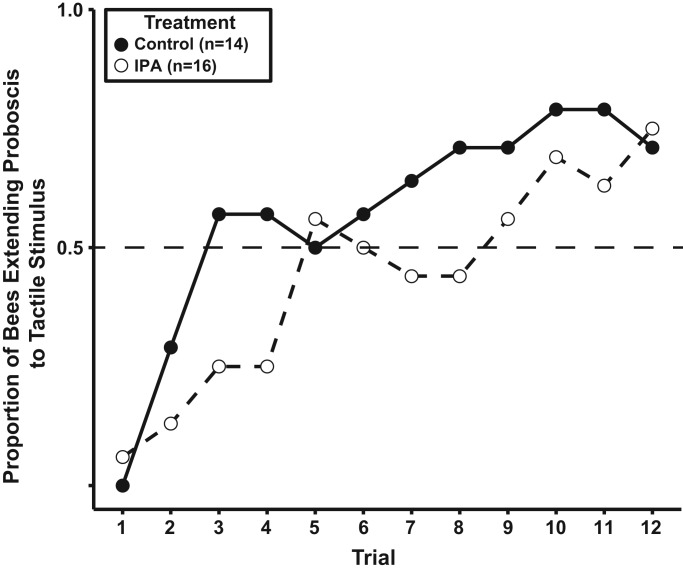
**Response of control and IPA exposed honey bee workers during proboscis extension assay.** Illustrated is the learned behavior of bees exposed to IPA (open circles, dashed line) and unexposed bees (filled circles, solid lines) during proboscis extension response conditioning assay. In this graph the Y-axis shows the proportion of individuals of each group that showed a conditioned response to the paired presentation of stimulus (antennal stroking) and reward (sugar water) over 12 learning trials (X axis). This is a conditioned response to the CS (not the proportion showing unconditioned responses to the US). Sample sizes for each group are provided in the boxed legend. Both IPA exposure was a significant predictors of response, with IPA treated bees being more likely to perform poorly when compared to control group (GEE, X^2^=7.37, d.f.=1, *P*=0.006) (see Supplemental data).

We selected the IPA dose to approximate what was described in past work on appetitive learning (Urlacher et al., 2010). Our method of calculating IPA dosages was different from previous studies and we have shown that our method produces comparable results since at an equivalent dosage level, IPA negatively affected the performance of worker honey bees establishing an appetitive association between test CS (antennal stroking) and US (sugar solution). Similar to the findings of Urlacher et al. (2010), by the third trial over 50% of those individuals in the Control group had formed the association, whereas only 25% of individuals exposed to IPA had done so. Unlike Urlacher et al. (2010), we continued training trials and found that this initial difference was reduced over time, so that by the twelfth learning trial an equivalent proportion of individuals from both groups had effectively acquired the association (Fig. 6). This finding suggests that IPA presentation may impair the rate of acquisition but not the ability to form appetitive associations.

Results of aversive learning under exposure to IPA show significant differences between some of the dose groups (1 and 10 SED vs 100 SED) and an overall negative association between learning performance and dose level (Figs 1 and 2). However, the learning differences are unlike appetitive learning, in that no dose of IPA results in significantly lower learning than control bees. Across worker groups there is a negative correlation between level of IPA presented during learning, and proportion of time spent on the safe side of the apparatus after the first shock (Fig. 1). From these findings we conclude that IPA presentation has a dose-dependent effect on honey bee aversive learning. This dose-dependent effect hypothesis explains the results that very low doses of IPA may improve learning, and this decreases at higher doses, making the control group intermediate (Fig. 2).

The response of honey bees to aggravating stimuli is under inhibitory control (Burrell and Smith, 1994, 1995; Giannoni Guzmán et al., 2014; Núñez et al., 1983; Ogawa et al., 2011). Pharmacological disruption of this control leads to increased sensitivity to negative stimuli (Giannoni Guzmán et al., 2014; Núñez et al., 1983; Tedjakumala et al., 2014). Greater sensitivity may enhance the salience of the punishment provided in our assay making it easier to establish learning at lower doses of IPA. Dose-specific effects are possible as high doses of IPA have been demonstrated to have an analgesic effect (see Nùñez et al., 1983), and this could make perception of electric shock, the aversive stimulus, difficult for the 100 SED group (Fig. 2). In addition, our IPA levels could also modulate neurophysiological response at the level of integration rather than perception. If doses of IPA differentially impact an individual's behavioral state (e.g. change in gene expression, see Li-Byarlay et al., 2014) this would elicit the context-specific effects observed, and could also explain the negative effect observed at the higher IPA doses.

An alternate explanation to dose-dependent effects for intermediate learning performance in the control group may be the differences in experience prior to testing. It is known that the effects of IPA are long lasting and involve mechanisms at the transcriptional level (Alaux and Robinson, 2007; Alaux et al., 2009). All our bees were collected as returning foragers, and they may represent a variable sampling of recent or remote past exposures to alarm pheromone. In contrast, our IPA exposure groups all experienced the signal at the moment of testing. Therefore, the observed response in the IPA exposed groups includes perception independent of bias, with the priming effect of alarm pheromone contributing to the observed variance. Future tests are required to differentiate between the previous experience and dose-specific effects hypotheses.

In contrast to workers, drones do not exhibit an IPA exposure-dependent modulation of aversive learning. Of further interest is that in the multiple odor presentation test drone, but not worker, learning was actually improved by presentation of our non-social odor cue (cineole, Fig. 5). Our results could be due to sex differences in perception thresholds to these odors. Cineole is known to be used by male Euglossine bees as a primary component of some of the odor bouquets used during mating displays (Schemske and Lande, 1984; Schiestl and Roubik, 2003). However, responses to the odor has served to separate cryptic species within the genus (Dressler, 1978), indicating taxonomic specificity of response to this chemical.

Another potential explanation may relate to differences in chemical ecology between the two sexes. There are examples of chemicals that typically are not part of the biology of an organism yet have a very strong influence on behavior (e.g. DEET and female mosquito behavior, and butyric acid and honey bee behavior, see Abramson et al., 2010). One possible reason for our findings is that cineole may interact with a sexually relevant signaling receptor in honey bee drones. Ultimately these findings underline the relevance of further research on each type of odor studied here.

Mechanisms through which the effect of IPA modulates learning include the possibility that IPA induces an arousal state which competes for neural resources needed by processes involved in the formation of associations. Work by Alaux et al. (2009) showed a down regulation of genes associated with brain metabolism after IPA presentation. Down regulation of brain metabolism has been previously shown to reduce neural activity in other model organisms such as rats and macaques (Du et al., 2008; Shmuel et al., 2006). In contrast, learning has been associated with increase in neural activity. This evidence supports a competitive trade-off in neural resources. If competition for neural resources is the driving factor, we would predict that impairment in the ability to form both appetitive and aversive associations would be evident. Our results do not demonstrate this, and we can state that IPA does not detrimentally influence aversive learning in all cases. Our results also show that the effect of IPA on learning has a degree of specificity, e.g. only affecting the rate of acquisition, not the ability to acquire appetitive associations, and exhibiting a dose dependency in aversive learning. These results are not consistent with a simple arousal-related trade-off model.

We conclude that modulatory effects of IPA as signals of social stress on an individual response are complex and context-dependent. The behavioral changes induced by exposure to IPA are dependent on the caste of the individuals. Future studies could expand on neural and molecular mechanisms of semiochemical influences on learning performance of individuals with different levels of social participation.

## MATERIALS AND METHODS

### Worker collections

Foragers were collected returning to their colonies in our research apiary at Gurabo Agricultural Research Station of the University of Puerto Rico in Gurabo, Puerto Rico. Collection was done between 08:00-17:00 h (Mattu et al., 2012). To collect individuals we blocked the colony entrance with a 6.32 mm^2^ wire mesh screen assuring no outflow of in-hive bees and a stalling of returning foraging bees. From each of the two colonies sampled, we collected individuals directly from the mesh using a collection vacuum modified so that suctioned bees were deposited directly into a collection tube (Model 5911, Type 1, 12V DC; BioQuip, Rancho Dominguez, CA). Immediately after collecting an adequate number of individuals we uncovered the entrance to restore worker flow, provided captured bees with 50% w/v sucrose solution, and transported them (<30 min commute) to our laboratory at the University of Puerto Rico, San Juan. Once here, they were transferred to a rearing cage (Bug Dorm, Model 1452, BioQuip, Rancho Dominguez, CA), provided with food (50% w/v sucrose solution) *ad libitum*, and kept overnight in a 34°C incubator.

### Drone collections

Due to seasonal changes in drone-brood availability we collected drones at the entrance and from inside colonies that were producing drone brood. Flight-age drones were collected at the entrance during peak flight period, 14:00 to 17:00 h in Puerto Rico (Galindo-Cardona et al., 2012). To collect these drones, we blocked the colony entrance with a queen excluder rather than the mesh used in worker collection (Benatar et al., 1995; see also Galindo-Cardona et al., 2012, 2015). In this way, we assured worker flow was not disturbed. Once the queen excluders were in place, all the colonies were sampled every 10-15 min. Drones on the queen excluders were collected with our modified vacuum. Multiple colonies were used to collect a genetically diverse sample representative of our population.

Drones collected in this way were returning from their practice or unsuccessful mating flights (Giray and Robinson, 1996; Dinges et al., 2013; Avalos et al., 2014). However, later in the season when drone production was greatly reduced, we collected drones from inside the colonies by opening the hives and extracting drones directly from the combs with the modified vacuum. Drones collected later in the season would be flight-age individuals (Galindo-Cardona et al., 2012, 2015). When an adequate number of drones were collected with either approach, they were taken to the laboratory for testing as with workers. Like workers, drones were also kept in rearing cages (Bug Dorm, Model 1452, BioQuip, Rancho Dominguez, CA), food was also provided for them *ad libitum*, and they were similarly kept overnight in a 34°C incubator.

### Proboscis extension response

To test the effects of isopentyl acetate (IPA) we first examined appetitive learning via a proboscis extension response assay (Bitterman et al., 1983; Menzel and Bitterman, 1983). Past studies demonstrated that IPA impaired the conditioned association of odor and reward (Becker et al., 2000; Urlacher et al., 2010, 2013). The negative effect of IPA on proboscis conditioning is also detectable in drones (Becker et al., 2000). As a test of our method (see below), we first examined whether the negative effect of IPA on appetitive learning can be repeated in our laboratory. To avoid potential confounds between the application of IPA and an olfactory conditioned stimulus (CS) we examined tactile, rather than olfactory, associations (Urlacher et al., 2010; Vergoz et al., 2007; Wang et al., 2016).

Worker bees that were previously collected were brought to our laboratory and immediately processed. Test bees were chilled on ice to anesthetize them and then placed in prepared bullet casings (0.32 Winchester Special, Browning Arms Company, Morgan, UT). These casings prevent any odor clinging, and allow easy cleaning for future use. Once secured, we restrained the bees in a manner which maximized researcher safety while still allowing them to comfortably extend and retract their proboscis (illustrated in Bitterman et al., 1983; and Menzel and Bitterman, 1983).

Restrained bees were provided with 50% w/v sucrose solution once they recovered from the analgesic effect of chilling. They were then placed in a dark container overnight to allow them to adapt. On the following day survivors (∼40-50%) were tested for responsiveness by presenting them with a filter paper dabbed in 50% w/v sucrose solution. Only those bees that readily extended their proboscis to feed were kept for testing. We would like to stress that in our experience, this prescreening measure is essential for good performance because it eliminates weak and slow responding bees while ensuring that all bees are highly motivated to respond to the unconditioned stimulus (US) (Abramson, et al., 2011).

Isopentyl acetate exposure followed Urlacher et al. (2010) with minor alterations. Our method of IPA presentation differed in that we did not dilute the compound in mineral oil, but rather used the compound density to calculate a volume of IPA that would provide equivalent vapor pressure to the maximum effective dose reported by Urlacher et al. (2010). Isopentyl acetate presentation was conducted in a sealed container whose volume (16.5 cm×15.5 cm×14 cm) was used together with the relative density of 98% IPA from Sigma-Aldrich^®^ to calculate the target IPA volume (29 µl) that would be equivalent to the maximum dosage presented by Urlacher et al. (2010). This target volume was deposited on a 1 cm^2^ piece of filter paper (Trans-Blot Paper^®^, 15×20 cm, 25, Bio-Rad Laboratories, Hercules, CA) which was placed with the bees inside the sealed container during the exposure period. Total duration of IPA exposure was 30 min for treatment groups, followed by a 30 min recovery period in a similar container but without the odor. Immediately after the 30 min recovery period, the proboscis response assay experiments began. Control groups were kept in the container for a full hour, but were not exposed to IPA during the first 30 min. Following the one hour containment period, the proboscis response assay experiments began.

A non-overlap procedure was used in which the CS terminated prior to the administration of the US (Abramson et al., 1999; Giurfa and Malun, 2004). The CS duration was 3 s and the US duration was 2 s. The CS consisted of three strokes of both antennas using a clean, stainless steel probe. Care was taken to ensure that an individual bee was not responding to a shadow of the probe (no bee responded to any shadow). The US was a 2 s feeding from a filter paper strip impregnated with 50% w/v sucrose solution and was presented manually by touching a subject's mouthparts with the filter paper strip and allowing the now extended proboscis to lick the filter paper. Each bee received a total of 12 training trials with a 10 min inter-trial interval. During each training trial, responses to the CS were recorded visually. If the bee extended its proboscis during the CS, a positive response was recorded. If the bee did not extend its proboscis during the CS a no response was recorded (Abramson et al., 1999; Giurfa and Malun, 2004).

The experimental design employed two groups. For one group, 14 bees were not exposed to IPA (Control Treatment). For a second group, 16 bees were previously exposed to IPA (IPA Treatment). To control for the effect of calendar variables per se, bees from both groups were trained daily.

Bees were run in daily ‘squads’ consisting of 3-7 bees. A trial was initiated by picking up a bee from its position in the squad and placing it in front of the experimenter. After a few moments, but never immediately upon placement, the CS was administered followed by the US. At the end of the US, the subject was returned to its position in the squad and the next bee was placed in front of the experimenter for its trial. When the last bee in the squad received its training trial, and the 10 min inter-trial interval (ITI) elapsed, the process was continued until each bee received 12 training trials. The results of this test (Fig. 6) are consistent with results of IPA effects on appetitive learning reported previously (Urlacher et al., 2010).

### Electric shock avoidance

**Fig. 7.**
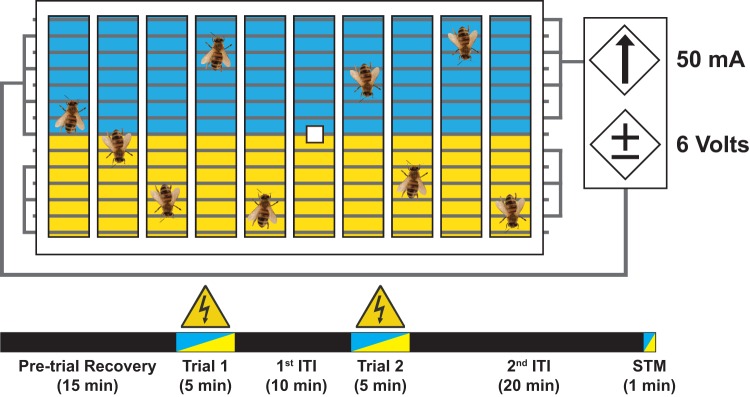
**Diagram of electric shock avoidance assay with corresponding time course.** This is a punishment with discrimination training paradigm that trains honey bees to avoid a color paired with a negative stimulus (mild shock). The figure illustrates the cassette of color background, intercalated grid, and individualized lanes. Additionally shown are the specific current and voltage settings for all tests. The figure also provides an illustrated breakdown of the 56 min learning challenge. In the illustrated time course, black bars denote periods of rest where no color or stimulus was experienced that occurred during pre-trial recovery from CO_2_ anesthesia, or at inter trial intervals (ITI). The two-toned portions of the bar illustrate the times when color was presented in association with shock (Trial 1, Trial 2, highlighted the electrical warning symbol) and a one minute presentation of colors without the shock as our short term memory (STM) test of the established association. Video records are available upon request.

For our aversive learning assay we tested the learning performance of both drones and foraging workers, since both demonstrate similar learning performance in this task (Dinges et al., 2013) (Fig. S1a, Fig. S2a, Fig. S3a, Fig. S4a). Bees were brought to the laboratory and kept in a dark incubator at 34°C overnight with food provided *ad libitum*. Following this adaptation period, we extracted a subset of nine individuals and placed them along individual lanes within our testing apparatus by first anesthetizing them with a 10-15 s inhalation of CO_2_ gas. At this time we also placed the odor cue (see below) on the sixth lane of the apparatus. The learning apparatus is primarily a ‘cassette’ made from a wire grid with individualized lanes cut from poster board, and top and bottom lids constructed from transparent Plexiglas™ (Fig. 7) (Agarwal et al., 2011).

The wire grid is divided into two halves so that the shock can be selectively administered by a power supply unit (BK PRECISION^®^ DC Power Supply Unit, Model#: 1610, Yorba Linda, CA, USA). On top of the grid we placed 10 individualized lanes cut into a 25 cm×15.5 cm section of white poster board (Wexford Poster Board, 56 cm×71 cm, Walgreen Co., Deerfield, IL, USA) which assured that no bee was capable of seeing or physically interacting with another. Lane dimensions were: 2 cm×13.5 cm×0.5 cm for the workers and 2 cm×13.5 cm×0.75 cm for the drones (Dinges et al., 2013). Drone size was accounted for by raising the lanes 0.25 cm using dark brown foam material (Foamy Sheets, 2 mm, 23 cm×29 cm, Walgreen Co., Deerfield, IL, USA) that was glued to and then cut along with the poster board. To prevent bees from escaping, while assuring color presentation, we placed layers of Plexiglas™ underneath the wire grid and on top of the cut poster board section. When assembled, we used Vaseline^®^ on the top Plexiglas™ sheet that prevented bees from walking on the material and escaping the shock. This apparatus was then placed on top of a computer monitor (DELL, Model #: E156FPc, Round Rock, TX, USA), where two colors: blue (Microsoft Paint default swatch, R: 0, G: 0, B: 255, Hue: 160, Sat: 240, Lum: 120) and yellow (R: 255, G: 255, B: 0, Hue: 40, Sat: 240, Lum: 120) were displayed. Color presentation was aligned so that one of the colors is paired with shock while the other was not, and colors where counterbalanced between sets of bees. Behavioral response was videotaped during the assay and target response measure (time spent on the safe side of the apparatus during acquisition trial) was later extracted from these video recordings.

The apparatus examined aversive learning using a discriminative punishment situation as first described by Agarwal et al. (2011). In this assay, bees freely walked upon the electrified grid, which is intercalated so as to create an open circuit. When the bees walked over the color associated with shock, they closed the circuit and received a mild shock (6 V, 50 mA). In this way, the bees associated one of the color cues with a negative stimulus (mild shock) which they must learn to avoid over two, 5 min, trial presentations and remember in a 1 min short term memory test. The total assay time including the pre-trial recovery and adaptation period is 56 min (Fig. 7). In this study we focused on aversive learning differences in the first trial i.e. the acquisition phase. This is important because in this and previous studies by the second minute of the first trial learning differences diminish, even across groups of bees exposed to ethanol (see Giannoni Guzmán et al., 2014) (Fig. S1a).

### Isopentyl acetate presentation and learning response

During the aversive assay, the presentation of IPA was continuous. We selected dosages that expanded on previously reported IPA concentrations and provided biologically relevant levels of IPA. We used the reported maximal per-bee mean quantity of IPA: 1500 ng in honey bees between 30 and 40 days old (Allan et al., 1987). We defined this quantity as a 1 sting-equivalent dose (SED) of IPA.

Using 1 SED we calculated the volume of 98% IPA from Sigma-Aldrich^®^ required to assure a stable evaporative release and diffusion of the chemical throughout our apparatus during our learning assay (0.3 µl 98% IPA). To achieve this, we again used the relative density of IPA and the combined volume of space in each of the individual lanes of our apparatus under the assumption that the porous poster material acting as visual divisors would pose no inhibition to the spread of the evaporated IPA gas phase.

To assay the effect of continuous presentation of IPA during aversive learning we utilized a dose response curve method. Using 1 SED as a base, we calculated corresponding volumes for 3 (0.9 µl 98% IPA), 5 (1.5 µl 98% IPA), 10 (3 µl 98% IPA), and 100 (30 µl 98% IPA) SEDs. This curve spanned presentations that were comparable to the three dose levels from Urlacher et al. (2010) (1, 3, and 5 SED), and added two more dose levels for testing (10 and 100 SED). Specifically we added the 10 SED level to assess the likelihood that during our standard 10 bee assay protocol all individuals responded by stinging; the 100 SED level was added to examine a quantity that would be more similar to a colony-wide alarm response such as during a predatory event in the field.

For each treatment group, we deposited target volumes of IPA on a 1 cm^2^ piece of filter paper and placed the filter paper on lane 6 of our apparatus at the same moment that we introduced the anesthetized bees. In this way diffusion of the odor would occur during the 15 min adaptation period, minimizing the possibility of a gradient forming at the time of testing. Using the aversive learning assay, we examined the responses of 9 honey bees simultaneously, with 4 replicates per treatment group. Hence 36 honey bees per group across five treatment and one control (no IPA) group were assessed (total *n*=216). From each group a subset of bees that did not interact with both sides of the apparatus were removed so that final worker sample sizes per group were: *n*=20 for the 0 SED, *n*=29 for the 1 SED, *n*=20 for the 3 SED, *n*=25 for the 5 SED, *n*=23 for the 10 SED, and *n*=29 for the 100 SED. Drone response was similarly examined but using a subset of the groups (control, 10 SED, and 100 SED dose levels). In total, 108 drones were assayed (9 per learning bout, 4 learning bouts per dose group). For drones, final sample sizes were: *n*=16 for the 0 SED, *n*=30 for the 10 SED, and *n*=29 for the 100 SED.

To prevent odor contamination, only one poster board divider was used per odor per caste. Following treatment, the piece of filter paper used for odor presentation was removed and deposited in a waste basket located in an area of the laboratory independent of where the assay was being conducted. Also, between treatments, all individual components of the apparatus were cleaned first with a Lysol^®^ solution, then with a 95% ethanol solution.

### Alternative odor presentation and learning response

We also assayed learning performance of workers and drones while under exposure to two other odors: cineole, potentially unbiased odor, commonly extracted from *Eucalyptus* leaves and used in learning assays (e.g. Behrends et al., 2007); and geraniol, the primary component in the compound emitted by the Nasonov's gland of honey bees as a positive social signal. The target volume of cineole (2.8 µl) was derived from previous studies which used the odor as an unbiased cue during appetitive associations in workers (e.g. Urlacher et al., 2010; Behrends et al., 2007). The volume of geraniol was derived from studies examining the physiology of the Nasonov's gland (Pickett et al., 1980; Williams et al., 1982). We calculated the per-bee proportion of geraniol to be 1800 ng, we then extrapolated the final presentation volume (2.3 µl) which assured that test bees were exposed to 100 bee-equivalent units of the compound.

The principal reason for using 100 bee equivalent units during presentation was to parallel the ecological function of Nasonov's gland emissions. The compound is a social signal that facilitates homing when foragers are returning to the colony, or during swarming (Pickett et al., 1981; Williams et al., 1982; Wilson, 1965). The experimental protocols for both odor presentation and learning assay were identical to that of the IPA presentation studies. The same number of individuals were assayed in drones (total *n*=144) and workers (total *n*=144), with 9 bees per learning bout and 4 bouts per odor assessed. Final sample sizes per group following removal of non-responding individuals were: *n*=20 control, *n*=22 cineole, *n*=17 geraniol, and *n*=29 IPA for the workers. For drones per group final sample sizes following removal of non-responders were as follows: *n*=16 control, *n*=30 cineole, *n*=32 geraniol, *n*=29 IPA.

### Data analyses

Quantification of behavior during proboscis extension reflex (PER) is done by recording binary response (extension, no extension) over a set of trials (12 in our case). Our data is therefore repeated measures of a binomial response, thus our analysis utilized logistic regression via generalized linear models. Specifically we used a generalized estimating equation (GEE; Zeger and Liang, 1986) which accounts for dependent responses (such as a time series) and allows for statistical inference of population response while accounting for within-subject correlations.

In the electric shock avoidance paradigm, analysis focused on the first 5 min training trial. The first 5 min of our aversive training assay corresponded to the acquisition of the aversive association, while the second 5 min trial has been shown by Agarwal et al. (2011) to parallel response expected in a reinforcement phase of learning, with bees that have acquired the association retaining maximal response (see detailed learning curves presented in Fig. S1a, Fig. S2a, Fig. S3a, Fig. S4a).

Individual honey bees differed in the time to interact (e.g. time to first error) with our aversive learning assay. To account for these differences we normalized each honey bee's response to the first time they experienced shock in our assay. We then calculated our response measure as the proportional amount of time spent on the safe side of the apparatus following this first shock. In this way our measure corrects for individual differences in activity.

Statistical analysis of dose groups examined differences across groups by applying a logit transformation to our response metric, proportion of time on safe side, followed by analysis via ANOVA and corresponding post hoc tests where significance was detected. Use of this transformation allowed for the application of standard parametric test on non-binomial, proportional measures, increasing our statistical power. For instances where differences across groups were observed, we conducted a correlation analysis between dose level and response.

In our experiment, the control group differs from the IPA exposed treatment groups. Specifically, while the treatment groups experience the social signal at the time of learning, control bees' response may be biased by past exposure to IPA. It is well know that IPA has transcriptional effects that last for days following exposure (see Alaux et al., 2007), thus the effect of past experience could increase the variance of the control group response depending on how recently our test bees may have been exposed to IPA. As we cannot control for this bias, we accounted for it in our analysis by conducting our correlation analysis with and without the control group (see below).

Past work has shown that a color bias can be present (Dinges et al., 2013). Our protocol counterbalances color presentation to mitigate this effect. To further account for it in our analysis, we included the color of the shock area as a co-factor with treatment in each of our statistical models. All statistical analyses were performed using the open source software R and corresponding packages (R Core Team, 2016); GEE analysis was conducted using the gee package from the R software suite (Carey, 2015).
